# Cost-Effectiveness Analysis of Influenza A (H1N1) Chemoprophylaxis in Brazil

**DOI:** 10.3389/fphar.2019.00945

**Published:** 2019-09-10

**Authors:** Luisa von Zuben Vecoso, Marcus Tolentino Silva, Mariangela Ribeiro Resende, Everton Nunes da Silva, Tais Freire Galvao

**Affiliations:** ^1^University of Campinas, Campinas, Brazil; ^2^Universidade de Sorocaba, Sorocaba, Brazil; ^3^University of Brasilia, Brasilia, Brazil

**Keywords:** cost-effectiveness, cost-utility, neuraminidase inhibitor, prophylaxis, influenza, Brazil, Unified Health System

## Abstract

**Background:** Oseltamivir and zanamivir are recommended for treating and preventing influenza A (H1N1) worldwide. In Brazil, this official recommendation lacks an economic evaluation. Our objective was to assess the efficiency of influenza A chemoprophylaxis in the Brazilian context.

**Methods:** We assessed the cost-effectiveness of oseltamivir and zanamivir for prophylaxis of influenza for high risk population, compared to no prophylaxis, in the perspective of Brazilian public health system. Quality-adjusted life years (QALY) and effectiveness data were based on literature review and costs in Brazilian real (BRL) were estimated from official sources and micro-costing of 2016’s H1N1 admissions at a university hospital. We used a decision-tree model considering prophylaxis and no prophylaxis and the probabilities of H1N1, ambulatory care, admission to hospital, intensive care, patient discharge, and death. Adherence and adverse events from prophylaxis were included. Incremental cost-effectiveness ratio was converted to 2016 United States dollar (USD). Uncertainty was assessed with univariated and probabilistic sensitivity analysis.

**Results:** Adherence to prophylaxis was 0.70 [95% confidence interval (CI) 0.54; 0.83]; adverse events, 0.09 (95% CI 0.02; 0.18); relative risk of H1N1 infection in chemoprophylaxis, 0.43 (95% CI 0.33; 0.57); incidence of H1N1, 0.14 (95% CI 0.11; 0.16); ambulatory care, 0.67 (95% CI 0.58; 0.75); hospital admission, 0.43 (CI 95% 0.39; 0.42); hospital mortality, 0.14 (CI 95% 0.12; 0.15); intensive care unit admission, 0.23 (95% CI 0.20; 0.27); and intensive care mortality, 0.40 (95% CI 0.29; 0.52). QALY in H1N1 state was 0.50 (95% CI 0.46; 0.53); in H1N1 inpatients, 0.23 (95% CI 0.18; 0.28); healthy, 0.885 (95% CI 0.879; 0.891); death, 0. Adverse events estimated to affect QALY in –0.185 (95% CI –0.290; –0.050). Cost for chemoprophylaxis was BRL 39.42 [standard deviation (SD) 17.94]; ambulatory care, BRL 12.47 (SD 5.21); hospital admission, BRL 5,727.59 (SD 7,758.28); intensive care admission, BRL 19,217.25 (SD 7,917.33); and adverse events, BRL 292.05 (SD 724.95). Incremental cost-effectiveness ratio was BRL –4,080.63 (USD –1,263.74)/QALY and –982.39 (USD –304.24)/H1N1 prevented. Results were robust to sensitivity analysis.

**Conclusion:** Chemoprophylaxis of influenza A (H1N1) is cost-saving in Brazilian health system context.

## Introduction

Influenza A (H1N1) prophylaxis with neuraminidase inhibitors is recommended by the World Health Organization (WHO), and health agencies of most developed and underdeveloped countries (World Health Organization, 2010; World Health Organization, 2019). Population at risk for influenza A complications includes pregnant and postpartum women, the elderly, children, indigenous people, immunosuppressed persons, health professionals, and long-term residents among others (Uyeki et al., 2018; Martinez et al., 2019). Influenza A accounted for 97% of the specimen circulating in the firsts months of 2019, of which 60% were influenza A (H1N1) 2009 pandemic (World Health Organization, 2019). Deaths associated with respiratory diseases from seasonal influenza accounts 300,000 to 650,000 annually (Iuliano et al., 2018). Higher burden of death is observed in less developed regions and in the elderly (Iuliano et al., 2018).

Complete efficacy data of neuraminidase inhibitors were published in 2014 and updated in 2016 (Jefferson et al., 2014a; Jefferson et al., 2014c; Heneghan et al., 2016). Before this effort, 60% of the patient data from phase III clinical trials have never been published; previous evidence could have been biased in favor of chemoprophylaxis (Jefferson et al., 2014b). Biases and conflicts of interests involved in research on influenza treatment and prevention translate into a need for studies on the drugs’ clinical performance vis-à-vis health systems’ financial investments (Jefferson et al., 2014b). Economic evaluations that take into consideration complete efficacy evidence are not available.

The efficiency of Influenza A (H1N1) chemoprophylaxis is also absent in in the Brazilian context, in which it is recommended and funded by the Ministry of Health (Brasil. Ministério da Saúde, 2017). Our objective was to assess the cost-effectiveness of influenza A (H1N1) chemoprophylaxis in the Brazilian public health system.

## Methods

### Target Population and Subgroups

Our target population were non-vaccinated or vaccinated for less than 15 days, people groups with high risk for influenza complications (the elderly, children, indigenous people, obese individuals, people with chronic diseases or immunodeficiency, pregnant or puerperal women), health care and laboratory workers exposed to samples or cases of influenza, and residents of nursing homes or inpatients during an outbreak (Brasil. Ministério da Saúde. Secretaria de Vigilância em Saúde, 2017).

### Setting and Location

The Unified Health System (*Sistema Único de Saúde*, SUS) is a public and universal health system (Paim et al., 2011). SUS is the public health sector responsible for primary care, access to medicines, immunization programs, complex services (cancer treatment and HIV/AIDS care), sanitary regulation, and sentinel surveillance, which monitors influenza by means of mandatory reports on flu syndrome and severe acute respiratory syndrome (World Health Organization, 2010). Access to these services has been largely improving since the system’s birth in 1988 (Paim et al., 2011). Despite this gradual improvement over the decades, SUS is systematically underfunded (Paim et al., 2011).

### Study Perspective

We adopted the SUS perspective and considered costs in the SUS context and excluded societal costs such as absence from work and patient personal costs. This involved costs for drug acquisition, health care services expenditure in cases of symptomatic diseases (ambulatory treatment, medical consultation, hospital admission, and procedures), and treatment of prophylaxis-related adverse events.

### Comparators

We assessed influenza A (H1N1) chemoprophylaxis in the aforementioned high-risk population, comparing oseltamivir and zanamivir prophylaxis with no prophylaxis.

Oseltamivir is an oral antiviral drug that inhibits the neuraminidase surface enzyme [Anatomical Therapeutic Chemical (ATC) code: J05AH02]. Its market availability was scientifically supported by experimentally infecting healthy subjects with influenza A and B (EPAR summary for the public, 2015). The drug effectively prevented influenza A infection after individuals were exposed to it (Jefferson et al., 2006), and was also able to reduce cases of symptomatic influenza within households (Dobson et al., 2015), as well as the time for alleviation of symptoms in infected adults. Oseltamivir significantly increased the incidence of nausea, vomit, and psychiatric events (Jefferson et al., 2006). Adults and children with more than 40 kg should take a 75 mg dose orally every 12 h, for 10 days. For children below this weight, the dosage is adjusted to 3–3.5 mg/kg for infants; 30 mg for children up to 15 kg; 45 mg, for over 15 to 23 kg; and 60 mg, until 40 kg (Brasil. Ministério da Saúde. Secretaria de Vigilância em Saúde, 2017).

Zanamivir is an antiviral selective neuraminidase inhibitor (ATC code: J05AH01) administered intranasally (Relenza, 2015). *In vitro* assays showed that low concentrations of the drug were able to inhibit influenza A and B neuraminidase. Symptom duration was reduced in healthy adults (median reduction 1.5 days; 1.0–2.5 days), but the mean time for symptom alleviation in elderly (>65 years) and in 5 or 6 year-old children was not significantly reduced. It has no documented benefits against non-febrile disease (body temperature < 37.8°C) (Relenza, 2015). Zanamivir is employed only in cases where oral oseltamivir is not feasible. Adults and children older than 5 years should receive two 5 mg inhalations per day for 10 days (Brasil. Ministério da Saúde. Secretaria de Vigilância em Saúde, 2017).

### Time Horizon and Discount Rate

We evaluated the outcomes of influenza A (H1N1) prophylaxis based on the duration of influenza infection, which is less than 21 days. No discount rate was applied.

### Choice of Health Outcomes

Quality-adjusted life years (QALY) was the primary outcome. Willingness-to-pay (WTP) threshold was considered to be 30,000 Brazilian real (BRL) per QALY (Soarez and Novaes, 2017). Prevented influenza A (H1N1) was also assessed, as a secondary outcome.

### Measurement of Effectiveness

#### Search Strategy

Data on oseltamivir’s and zanamivir’s effectiveness in preventing symptomatic flu and its complications was gathered from search on the literature held on March, 2017. The following search strategy was employed in the MEDLINE (via PubMed) database: (oseltamivir OR tamiflu OR zanamivir OR relenza OR “neuraminidase inhibitors”) AND (H1N1 OR influenza) AND (“clinical trial”[Filter] OR “systematic”[Filter] OR cost OR economic). The same strategy was adjusted to Embase, Scopus, and Cochrane Library databases. Additional searches were performed to ascertain effectiveness and cost data in the Brazilian scenario. Results were imported to Covidence (www.covidence.org) for identifying duplications; pair selection was performed by two independent researchers. Systematic reviews, randomized clinical trials, and observational studies were included.

Complementary non-systematic searches were performed in order to gather specific data on prevalence, hospitalization, death in hospital, and other variables included in the model. Information was also collected from SUS electronic systems whenever needed. When estimates from different studies were available, random-effect meta-analysis was performed using *Stata* (version 14.2).

#### Quality Assessment of Included Studies

We assessed the quality of all the included studies using standard instruments: A MeaSurement Tool to Assess systematic Reviews (AMSTAR 2) for systematic reviews (Shea et al., 2017), Newcastle–Ottawa scale for cohort and case–control studies (Wells et al., 2000), and the Joanna Briggs Institute checklist for prevalence studies (Munn et al., 2015).

### Estimating Resources and Costs

Costs of oseltamivir and zanamivir acquisition were obtained from 2016 purchase data, provided by the Brazilian Ministry of Health, using information made available by the Pharmaceutical Assistance Department. Health care assistance costs were obtained from the SUS reimbursement system (http://sigtap.datasus.gov.br/tabela-unificada/app/sec/inicio.jsp). We considered the dosage and administration according to Brazilian guidelines (Brasil. Ministério da Saúde. Secretaria de Vigilância em Saúde, 2017).

Health expenditures were obtained from micro-costing of all inpatients admitted in 2016 for H1N1 treatment at the Clinics’ Hospital of the University of Campinas, Campinas, São Paulo — a 400-beds high complexity hospital.

### Currency, Price Date, and Conversion

Costs were calculated in BRL acquisitive value in 2016. Costs gathered from the literature from previous years were corrected to 2016 using the Brazilian consumer’s price index (*Índice de Preços ao Consumidor*, IPCA) (https://ww2.ibge.gov.br/home/estatistica/indicadores/precos/inpc_ipca/defaultinpc.shtm). The obtained incremental cost-effectiveness ratio (ICER) was converted to United States dollars (USD) using the exchange rate for July 1^st^, 2016 provided by Brazil’s Central Bank (1 USD = 3.229 BRL) (https://www4.bcb.gov.br/pec/taxas/ingl/ptaxnpesq.asp?id=quotations).

### Choice of Model

**Figure 1:**
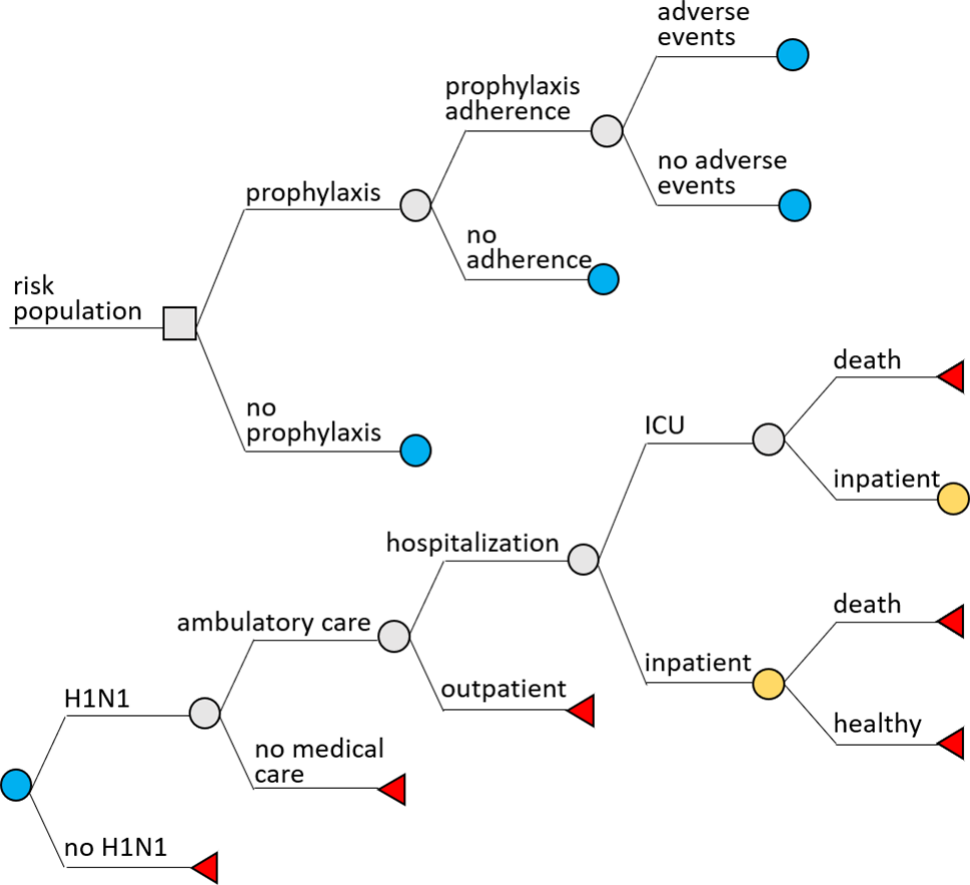
Decision-tree model adopted in the analysis.

TreeAge Pro 2018 (R.2.0) software was used to build a decision-tree model. Two scenarios were considered: chemoprophylaxis and no chemoprophylaxis. In both scenarios, the following probabilities were assessed: H1N1 infection, ambulatory care, hospital admission, intensive care admission, patient discharge, and death. In the prophylaxis scenario, we included adherence to prophylaxis and incidence of adverse events (**Figure 1**).

Costs for outcomes were calculated considering that all flu cases were influenza A (H1N1) type; half-cycle correction was used to calculate costs for cases with death as the final outcome. Clinical plausibility was evaluated by an infectious disease specialist doctor, who was part of the research team (MRR) and had experience in influenza management.

### Assumptions

We considered that all symptomatic patients would seek outpatient care. Hospital admission was assumed as a probability for those seeking ambulatory care, and admission to the intensive care unit as a probability for people admitted to the hospital. Death was assumed as possible only for people admitted to the hospital or to the intensive care. Subjects who did not develop flu were considered healthy. No sequelae or late effects of influenza were considered.

### Analytical Methods

Uncertainties of the model were estimated according to variations in the adopted parameters. A tornado diagram of minimum and maximum values was used for univariate sensitivity analysis. Sensitivity-enhancing model parameters were chosen for best- and worst-case scenario analyses.

Probabilistic sensitivity analysis was performed using Monte Carlo, employing a 10,000 simulation count and threshold analysis to identify the maximum cost of the technology, all other parameters unchanged (*ceteris paribus*). We used variables as distribution; beta distribution was adopted for probabilities and outcomes, gamma for costs, and log-normal for relative risk (Bilcke et al., 2011).

### Ethics Approval Statement

The study was approved by the University of Campinas Ethics Committee, report number 2,357,158 issued on October, 30^th^ 2017. The study was exempt from consent procedure, once patient data would be from medical records.

## Results

### Study Parameters

#### Effectiveness Data

Probabilities of efficacy adopted are described in **Table 1**.

**Table 1:** Probabilities of outcomes, distribution parameters adopted in the analytical model, and sources.

Variable	Effect (95%CI)	Distribution parameters^a^	Source	Quality of evidence
Prophylaxis adherence	0.70 (0.54; 0.83)	α = 26 β = 11	Proportion of health professionals that completed post-exposure prophylaxis during 2009 pandemic in a hospital in Melbourne, Australia (Upjohn et al., 2012)	5/9 ^b^
Adverse events incidence	0.09 (0.02; 0.18)	Mean = 0.09 SD = 0.06	Sum of risk differences for significant adverse events (headache, nausea, and psychiatric events) (Jefferson et al., 2014a)	High-quality review ^c^
Prevention of H1N1 with chemoprophylaxis	0.43 (0.33; 0.57)^d^	µ = −0.84 σ = 0.14^e^	Meta-analysis of 7 clinical trials for the prophylaxis with oseltamivir or zanamivir in the general population (Jefferson et al., 2014a)	High-quality review ^c^
H1N1 in risk population	0.14 (0.11; 0.16)	Mean = 0.14 SD = 0.02	Meta-analysis of 20 incidence studies on febrile acute respiratory syndrome in households (Lau et al., 2012)	Critically-low quality review ^c^
Ambulatory care	0.67 (0.58; 0.75)	Mean = 0.67 SD = 0.04	Meta-analysis comprising 38 studies on the incidence of symptoms after experimental infection with influenza (Carrat et al., 2008)	Critically-low quality review ^c^
Hospital admission	0.43 (0.39; 0.42)	α = 1,911 β = 2,809	Proportion of hospital admission among confirmed H1N1 cases in 2010, Parana, Brazil (Lenzi et al., 2012)	8/10 ^f^
Death in hospital	0.14 (0.12; 0.15)	α = 258 β = 1,653	Mortality in hospital among confirmed H1N1 cases in 2010, Parana, Brazil (Lenzi et al., 2012)	8/10 ^f^
Intensive care unit admission	0.23 (0.20; 0.27)	α = 148 β = 484	Proportion of intensive care admission among inpatients of the Clinics’ Hospital of the University of Sao Paulo during 2009 pandemic (Calmona, 2013)	8/9 ^b^
Death in intensive care unit	0.40 (0.29; 0.52)	α = 25 β = 38	Mortality among H1N1 patients in 11 intensive care units during 2009 pandemic, Parana, Brazil (Duarte et al., 2009)	8/10 ^f^

^a^beta distribution. ^b^Joanna Briggs Institute checklist. ^c^AMSTAR 2. ^d^relative risk. ^e^log-normal distribuiton. ^f^Newcastle-Ottawa scale. CI, confidence interval; SD, standard deviation.

Prophylaxis adherence was considered to be 70%, according to adherence data from health professionals exposed to H1N1 virus during the 2009 pandemic (Upjohn et al., 2012). The incidence of adverse events among those who adhered to the prophylaxis was estimated as 9%, based on the incidence of headaches, nausea and psychiatric events—the most frequent and significant adverse events (**Appendix A**).

Risk of H1N1 infection in the high-risk population was considered to be 14%, based on the incidence of symptomatic infection among households which had contact with infected patients (Lau et al., 2012). The relative risk of H1N1 infection with prophylaxis was considered to be 0.43 [95% confidence interval (CI) 0.33; 0.57], according to meta-analysis for the prophylaxis with the antivirals (Jefferson et al., 2014a) (**Data Sheet S1**). Since scientific evidence showed no efficacy for preventing complications (a proxy for seeking for medical care), hospital or intensive care admission and death from influenza (Jefferson et al., 2014a; Jefferson et al., 2014c; Heneghan et al., 2016), these variables had the same probability in both prophylaxis and no prophylaxis branches: the probability of seeking medical care (ambulatory care) was 0.67, the incidence of symptomatic illness after experimental influenza infection (Carrat et al., 2008), assuming that all people who developed symptoms would seek medical care.

Incidence of hospital (43%), and intensive care (23%) admission, hospital (23%), and intensive care mortality (40%) were based on Brazilian studies held during the 2009–2010 pandemics (Duarte et al., 2009; Lenzi et al., 2012; Calmona, 2013). Complete quality assessment of studies that provided data to the model are available at **Data Sheet S2**.

#### Utility

QALY for H1N1 infections managed in outpatient services was 0.50 and those admitted to hospital or intensive care was 0.23 based on a study with patients infected with H1N1 during the 2009 pandemic (Hollmann et al., 2013). Adverse events reduced QALY in 0.195 (**Appendix A**). The QALY for the healthy state was 0.885, the mean QALY measured in two population-based Brazilian studies (Zimmermann et al., 2016; Silva et al., 2017). QALY for death was 0 (**Table 2**).

**Table 2:** Utilities considered in the model.

Health state	QALY (95%CI)	Mean (SD)^a^	Source	Quality of evidence
H1N1 outpatient	0.50 (0.46; 0.53)	0.50 (0.02)	QALY for outpatients infected with H1N1 during the 2009 pandemic, Spain (Hollmann et al., 2013)	7/10 ^b^
H1N1 inpatient	0.23 (0.18; 0.28)	0.23 (0.03)	QALY for inpatients infected with H1N1 during the 2009 pandemic, Spain (Hollmann et al., 2013)	7/10 ^b^
Adverse events	−0.195 (−0.290; −0.050)^c^	−0.195 (0.121)	Reducion in QALY (Lindner et al., 2009; Araujo et al., 2014) weighted to the incidence of each adverse event (Jefferson et al., 2014a) (Appendix A)	Low quality^d^
Healthy	0.885 (0.879; 0.891)	0.885 (0.003)	Weighted mean QALYy assessed by Brazilian population-based studies (Zimmermann et al., 2016; Silva et al., 2017)	8/9 ^e^
Death	0	0	-	

^a^beta distribution. ^b^Newcastle-Ottawa scale. ^c^reduction on QALY due to adverse events. ^d^data from previous economic evaluation which used multiple sources. ^e^Joanna Briggs Institute checklist (both studies had this score). QALY, quality-adjusted life years; SD, standard deviation.

#### Costs

Cost with prophylaxis was BRL 39.42, based on average expenditure of Brazilian Ministry of Health with the antivirals (**Appendix B**). Treatment of prophylaxis’ adverse cost BRL 292.05, calculated from the cost of each main adverse event (headache, nausea, and psychiatric event) weighted to each adverse event incidence (**Appendix A**).

Outpatient care cost BRL 12.47 according to SUS reimbursement for an urgent care consultation. Cost of hospital admission was estimated in BRL 5,727.59 and for intensive care, BRL 19,217.25 (**Table 3**).

**Table 3:** Costs included in the model, in Brazilian real.

Cost item	Mean (SD)^a^	Source
Chemoprophylaxis	39.42 (17.94)	Brazilian Ministry of Health’s costs with oseltamivir and zanamivir acquisition, 2016 (Appendix B)
Ambulatory care	12.47 (5.21)	Procedure code 03.01.06.002-9 — urgent care with 24-hour observation, with specialized care (SIGTAP database)^b^
Hospitalization	5,727.59 (7,758.28)	Micro-costing of inpatients with H1N1 in 2016 at Clinics’ Hospital of the University of Campinas
Intensive care unit	19,217.25 (7,917.33)	Micro-costing of intensive care unit in patients with H1N1 in 2016 at the Clinics’ Hospital of the University of Campinas
Adverse events	292.05 (724.95)	Cost of each event in proportion to incidence (Appendix A)

^a^gamma distribution. ^b^available from: http://sigtap.datasus.gov.br/tabela-unificada/app/sec/inicio.jsp; SD, standard deviation.

### Incremental Costs and Outcomes

The prophylaxis scenario was undominated, while no prophylaxis was absolutely dominated (**Table 4**). The incremental cost of prophylaxis was BRL –54.45, and QALY increased 0.013, resulting in an ICER of BRL –4,080.63 per QALY (USD –1,263.74/QALY). For the secondary outcome prevention of H1N1 infection, incremental QALY was 0.055, and ICER was BRL –982.39 per prevented case (USD –304,24/prevented H1N1).

**Table 4:** Costs, effectiveness and incremental cost-effectiveness ratio (ICER) of prophylaxis compared to no prophylaxis.

Scenario	Cost (BRL)	QALY	Prevented H1N1
Prophylaxis	230.83	0.832	0.915
No prophylaxis	285.29	0.819	0.860
Incremental	−54.45	0.013	0.055
ICER (BRL)		−4,080.63	−982,39
ICER (USD)		−1,263.74	−304.24

QALY, quality-adjusted life years; BRL, Brazilian real (1 USD = 3.229 BRL); USD, United States dollar.

### Characterizing Uncertainty

#### Univariate Sensitivity Analysis

**Figure 2:**
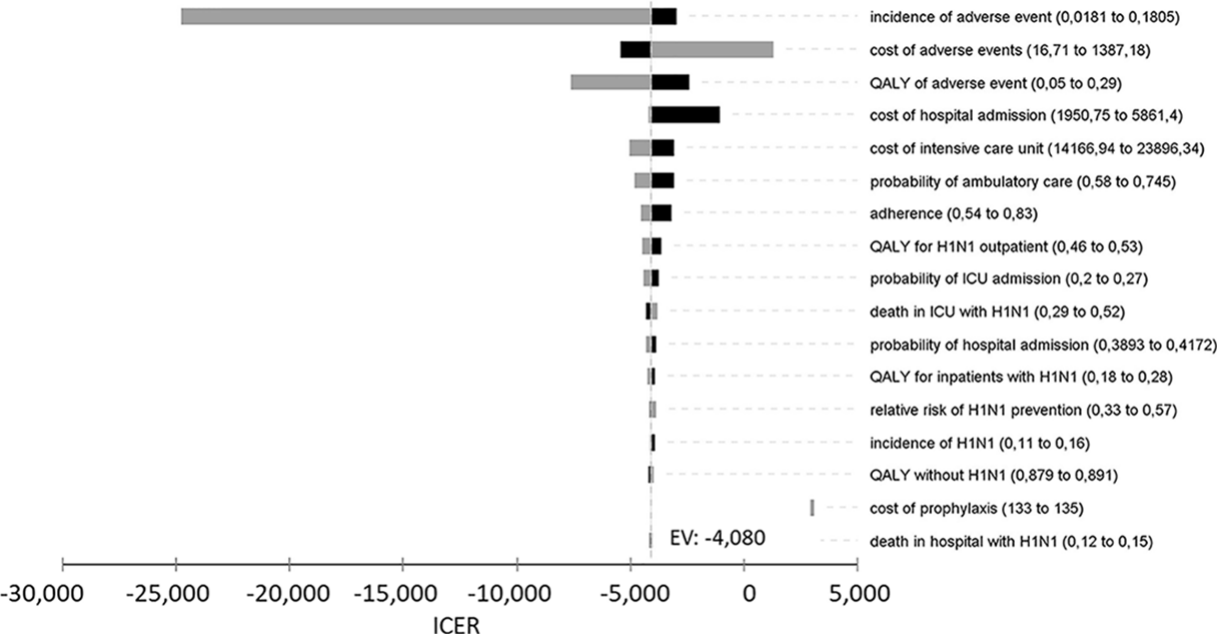
Univariate sensitivity analysis of incremental cost-effectiveness ratio (ICER) of chemoprophylaxis compared to no prophylaxis. QALY, quality-adjusted life years; ICU, intensive care unit.

The tornado-diagram sensitivity analysis demonstrated the robustness of our model when using expected intervals for each variable (**Figure 2**). None of the variables changed the cost-effectiveness profile of the technology given the adopted WTP threshold (BRL 30,000.00/QALY). The ICER remained robust after best- and worst-case scenario analysis with highest impact variables in the tornado (**Table 5**). Threshold analysis led to BRL 134.00 limit for chemoprophylaxis cost-effectiveness.

**Table 5:** Incremental cost-effectiveness ratio for best- and worst-case scenarios (variables with the highest impact in the univariate sensitivity analysis).

Variable	Best-case scenario	Worst-case scenario
Incidence of adverse event	−24,783.28	−2,956.06
Cost of adverse events	−5,435.36	1,307.65
Utility reduction in case of adverse events	−7,650.05	−2,383.32
Cost of prophylaxis	−5,399.30	−2,249.13

## Probabilistic Sensitivity Analysis

**Figure 3:**
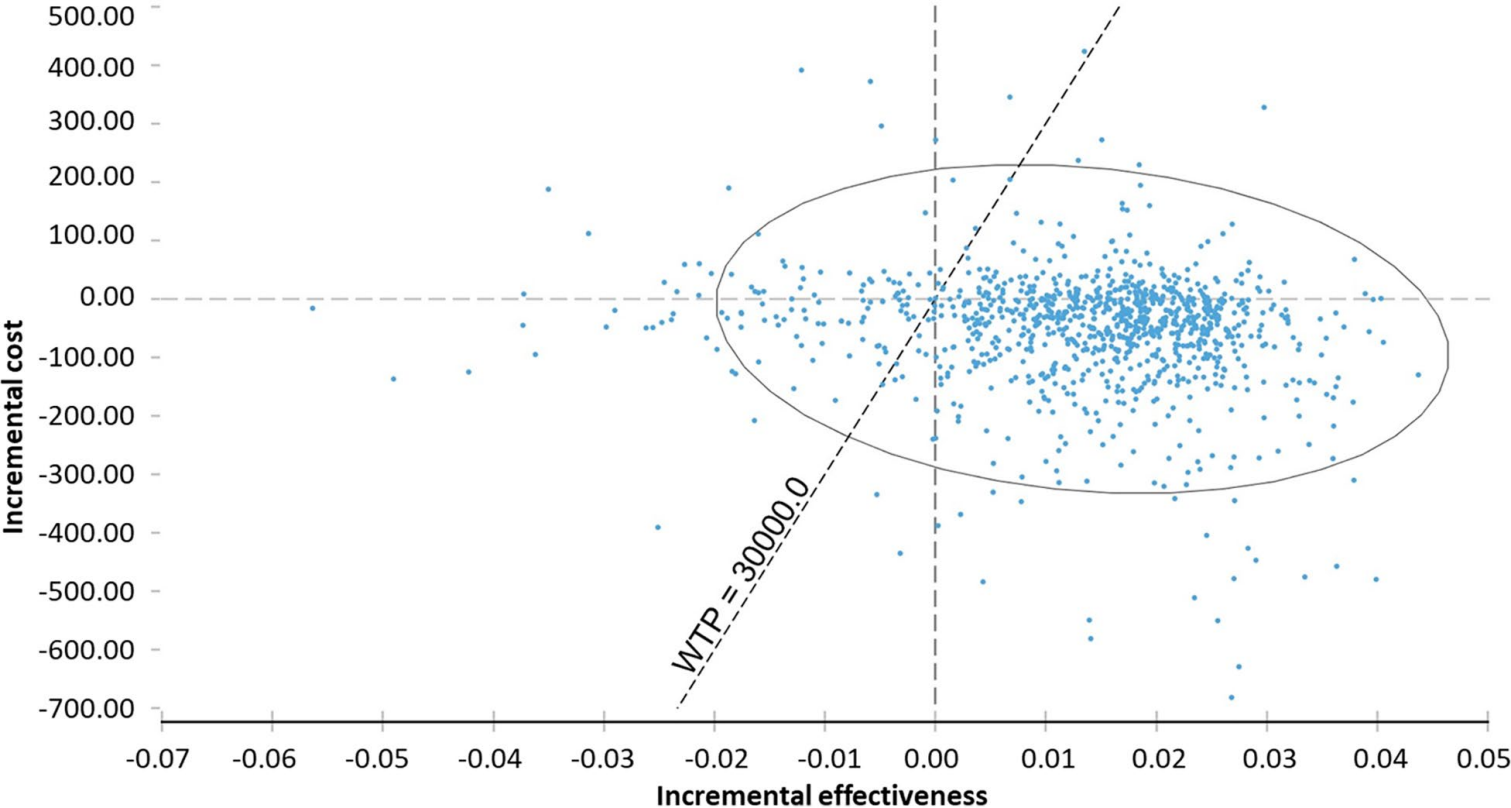
Probabilistic sensitivity analysis of incremental cost-effectiveness ratio of chemoprophylaxis compared to no prophylaxis. WTP, willingness to pay.

In the probabilistic sensitivity analysis, 68% of ICER would be in fourth quadrant (higher effectiveness and lower cost) and 18% of ICER, in first quadrant (higher cost and effectiveness). The probability of the technology being under the WTP threshold (BRL 30.000/QALY) was 97.9% (**Table 6**, **Figure 3**).

**Table 6:** Probabilities (p) of incremental cost-effectiveness ratio (ICER) in each quadrant according to 10,000 Monte Carlo simulations, chemoprophylaxis versus no prophylaxis.

Quadrant	Incremental effect	Incremental cost	ICER	n	p
IV	>0	<0	Superior	6,849	0.6849
I	>0	>0	<30.000	1,793	0.1793
III	<0	<0	>30.000	153	0.0153
I	>0	>0	>30.000	57	0.0057
III	<0	<0	<30.000	749	0.0749
II	<0	>0	Inferior	399	0.0399

## Discussion

H1N1 prophylaxis compared to no prophylaxis was cost-saving in the context of the Brazilian health system for both QALY and prevention of H1N1 outcomes. The mean cost calculated from micro-costing are aligned to previous Brazilian studies that estimated the cost of hospital admission to influenza A (H1N1) (Silva et al., 2012).

The chemoprophylaxis reduces the cost and the increases the effectiveness of influenza A (H1N1) prevention. Its effect on QALY (0.013), however, may be clinically irrelevant. In any case, preventing a single influenza A (H1N1) case by means of prophylaxis could save nearly BRL 1,000. At the same time, Brazil has no official WTP threshold (Soarez and Novaes, 2017). Whether present represents a cost-effective alternative is subject for debate. Effects of neuraminidase inhibitors on prophylaxis came from clinical trials in which exposure to H1N1 and treatment onsets were highly controlled. The effectiveness for chemoprophylaxis is limited to strict conditions according to a mathematical modelling and computer simulations, and stockpiling for this situation is questioned (Parra-Rojas et al., 2018). Despite a protocol to start the drug in the first 24 hours post-exposure, pragmatic clinical trial revealed late initiation of oseltamivir at the hospital setting without reduction of clinical failures among the assessed groups (Ramirez et al., 2018). This potentially unrealistic efficacy data may have inflated the effects of prophylaxis.

We obtained influenza prevention efficacy data from systematic reviews carried out as the offspring of a Cochrane Collaboration and The BMJ campaign to obtain complete clinical trials data from Roche, the drug manufacturer. The campaign’s efforts led to the publication of the systematic review in 2014; it was then updated in 2016, with no changes in the results (Jefferson et al., 2014a; Jefferson et al., 2014c; Heneghan et al., 2016). Sixty percent of patient data in phase III clinical trials had never been published, suggesting that previously-published research was biased in favor of the technology (Jefferson et al., 2014a). Publication bias was reduced once all clinical trials with the drugs were taken into consideration in such efforts (Jefferson et al., 2014a; Jefferson et al., 2014c; Heneghan et al., 2016).

Some of our probabilities were based on data from studies held during the 2009 influenza A (H1N1) pandemic, a period marked by greater virulence of influenza in Brazil and worldwide (Ministério da Saúde, 2012). In 2009, cases of severe acute respiratory syndrome in Brazil reached more than 44 per 100,000 inhabitants; later in 2010, its occurrence decreased to 4.6 cases per 100.000 inhabitants, finally reaching 2.5 in 2011. Influenza vaccine has been part of programmed vaccination for the elderly (>65 years of age) since 1999, and its use was expanded in 2010 to people >60 years of age. In 2011, pregnant women, children between six months and two years of age, indigenous people and health workers were included; since then, vaccine coverage has hovered above 80% (Ministério da Saúde, 2012). The probabilities adopted in our model led to more severe consequences for influenza, favoring the prophylaxis performance. We assumed that all patients with symptoms would seek for medical care, therefore we did not consider “out-of-pocket” expenses in cases that patients would treat themselves without seeking for medical consult, as did an economic study of dengue in Brazil (Godoi et al., 2018). Adherence to prophylaxis was based on health professionals during the 2009 pandemic, period with greater concern about infection. Such assumptions and use data from the pandemia brought to a more conservative scenario that probably does not reflect the current scenario, where more people vaccinated and greater herd immunity is granted. Deterministic and probabilistic sensitivity analysis attested robustness of cost-effectiveness when probabilities of infection, hospital admission, and death by H1N1 ranged, partially circumventing these limitations.

The primary outcome of our study was based on QALY from the Spanish context, due to lack of utility data for influenza in Brazil. QALY for healthy state was based in Brazilian population data (Zimmermann et al., 2016; Silva et al., 2017). We evaluated the prevention of influenza as a secondary outcome, which does not involve population perception and favored prophylaxis. The Brazilian protocol for influenza states that chemoprophylaxis should be administered to non-vaccinated or vaccinated for less than 15 days people (Brasil. Ministério da Saúde. Secretaria de Vigilância em Saúde, 2017). Data on the effectiveness of the antiviral drugs segregated by vaccination status were not available for a specific analysis of the target-population not under the vaccine’s effect. Influenza vaccination showed to reduce healthcare utilization in the elderly (Doyon-Plourde et al., 2019), as well as antibiotic usage in health adults (Buckley et al., 2019). While maintaining consistency with the national guideline, ignoring the effect of vaccination in our model may have favored the need and effectiveness of the chemoprophylaxis.

Our study is similar to previous health economic evaluations on influenza chemoprophylaxis, which also adopted a decision-tree model with a time horizon shorter than one year and favored the prophylaxis. In the Canadian health system, post-exposure prophylaxis in institutionalized and vaccinated elderly was dominant for preventing influenza-like illnesses when compared to no prophylaxis (Risebrough et al., 2005). This evaluation was based on three alternatives – prophylaxis with amantadine, prophylaxis with oseltamivir and no prophylaxis – and predicted viral resistance and adverse effects on the amantadine branch, influenza-like illnesses, complications, death, survival, and treatment in hospital or institution (Risebrough et al., 2005). The research was sponsored by oseltamivir manufacturer, F. Hoffmann-La Roche.

In the United Kingdom, post-exposure prophylaxis for inter-family contacts was probably cost-effective in the context of the National Health System, considering 2002’s cost data (Sander et al., 2006). The model compared prophylaxis to no prophylaxis with or without oseltamivir treatment in the case of symptomatic influenza, and predicted complications, outpatient care, hospital admission, recovery and death, and assessed QALY and avoided cases of influenza-like illness. Probabilistic and sensitivity analysis attested the robustness of the model (Sander et al., 2006). The study was also sponsored by F. Hoffmann-La Roche, and the last author was an employee of the company.

United States analysis of post-exposure prophylaxis with oseltamivir in children up to 12 years was cost-effective in the perspectives of society and the payer, with 2008’s costs (Talbird et al., 2009). The model compared prophylaxis to no prophylaxis and predicted development of influenza, hospital admission, outpatient care, death, and survival (Talbird et al., 2009). The research was commissioned by Roche, and the last author was its employee.

The National Health System in the United Kingdom funded a systematic review about efficacy and effectiveness of seasonal and post-exposure prophylaxis, with subsequent analysis of cost-effectiveness using amantadine, oseltamivir, and zanamivir in vaccinated and non-vaccinated individuals (Tappenden et al., 2009). Six subgroups were considered: children, adults and elderly, in high-risk or healthy states, using cost data for 2006. Influenza-like illnesses, search for outpatient care, antiviral treatment, complications, death, and survival were considered in the analysis (Tappenden et al., 2009). The model predicted adverse effects to amantadine, vaccination, and prophylaxis abandonment ranging from 1.3% to 14.7%. Post-exposure prophylaxis was under 30,000.00 British pounds/QALY for non-vaccinated children and the elderly. None of these economic assessments considered herd immunity, adverse events of the studied drugs, and the complete efficacy data with lower risk of publication bias (Jefferson et al., 2014a; Jefferson et al., 2014c; Heneghan et al., 2016).

## Conclusion

Post-exposure prophylaxis for influenza A (H1N1) is cost-saving in the context of the Brazilian public health system. Current Brazilian guidance for influenza A (H1N1) prevention is supported by the findings, but a lack of national efficacy and effectiveness data is noticed. Both oseltamivir and zanamivir are already incorporated for this purpose, changes to current guidelines are unnecessary.

## Ethics Statement

Our study was approved in October 30, 2017 by Unicamp Ethics Committee (2.357.158) de 30/10/2017, certificate number 78192417.0.0000.5404. The study was exempt from consent procedure, once data was from patient medical records.

## Author Contributions

LV, TG and MS designed the work, LV collected the data, LV and TG did the analyses and drafted the work, MS, ES and MR interpreted the data and revised the work critically. All authors approved the version to be published and agree to be accountable for all aspects of the work.

## Funding

The study was funded by HAOC – PROADI SUS. Nonfinancial support was provided by Clinics’ Hospital and Faculty of Pharmaceutical Sciences of the University of Campinas.

## Conflict of Interest Statement

The authors declare that the research was conducted in the absence of any commercial or financial relationships that could be construed as a potential conflict of interest.

The handling editor and reviewer BG declared their involvement as co-editors in the Research Topic, and confirm the absence of any other collaboration.

## Appendix

### Appendix A Cost and Utility of Adverse Events

We used the risk difference of each significant adverse event reported in the systematic review (Jefferson et al., 2014a) to calculate the probabilities of adverse events.

**Table:**

Event	Risk difference, % (95%CI)	Weight (%)	Costs		QALY	
			Raw	Weighted	Raw	Weighted
Headache	3.15 (0.88; 5.78)	33.7	16.71^a^ (Araujo et al., 2014)	5.625	−0.050 (Araujo et al., 2014)	−0.017
Nausea	5.15 (0.86; 9.51)	55.0	235.06^a^ (Araujo et al., 2014)	129.33	−0.290 (Araujo et al., 2014)	−0.160
Psychiatric^b^	1.06 (0.07; 2.76)	11.3	1,387.18^c^ (Lindner et al., 2009)	157.10	−0.167 (Lindner et al., 2009)	−0.019
Total	9.36 (1.81; 18.05)	100.0	–	292.05^d^	−	−0.1953

^a^2014’s costs corrected to 2016. ^b^“suspected serious psychotic/suicidal adverse events (including hallucination, psychosis, schizophrenia, paranoia, aggression/hostility and attempted suicide)” (Jefferson et al., 2014a). ^c^2009’s costs corrected to 2016. ^d^Standard deviation = 736.34. QALY, quality-adjusted life years.

### Appendix B Expenditure With Acquisition of Oseltamivir and Zanamivir by the Pharmaceutical Services Department, Brazilian Ministry of Health in 2016

It is worth noting that we did not consider stockpiling costs and loss due to product expiration, since such data was unavailable. This would be important information for calculating the total cost of chemoprophylaxis.

**Table:**

Medicine	Unity	Unity price	Prophylaxis price^a^	Expenditure^b^	Weighted price of prophylaxis (BRL)
Oseltamivir 30 mg	Capsule	2.18	21.78^c^	3,036,670	2.58
Oseltamivir 45 mg	Capsule	3.27	32.70	2,578,500	3.28
Oseltamivir 75 mg	Capsule	4.29	42.92	20,057,500	33.53
Zanamivir 5 mg	Kit	63.92	63.92^d^	1,000	0.02
**Total**				25,673,670	**39.42**

BRL, Brazilian real; ^a^standard deviation = 17.94. ^b^expenditure in BRL from 01/01/2016 to 08/23/2017. ^c^minimum value adopted on the univariate sensitivity analysis. ^d^maximum value adopted on the univariate sensitivity analysis.
