# Supplementary material for: Cost-Effectiveness Analysis of Influenza A (H1N1) Chemoprophylaxis in Brazil
Source: Front Pharmacol. 2019 Sep 10;10:945. doi: 10.3389/fphar.2019.00945 (PMC6749104; doi:10.3389/fphar.2019.00945)
Supplement: Supplementary file 1 [file DataSheet_1.docx]

Supplementary file 1. Prevention of influenza by the prophylaxis with oseltamivir or zanamivir in general population: raw data from clinical trials (a) (based on Jefferson et al., 2014), and random effect meta-analysis (b)

a

| Study | Prophylaxis | | Control | | antiviral |
| --- | --- | --- | --- | --- | --- |
|  | events | total | events | total |  |
| 167-101 | 3 | 160 | 6 | 156 | zanamivir |
| NAI30034 | 4 | 1678 | 23 | 1685 | zanamivir |
| NAIA3004 | 15 | 240 | 23 | 249 | zanamivir |
| NAIA3005 | 11 | 553 | 34 | 554 | zanamivir |
| WV15673/WV15697 | 30 | 1040 | 36 | 519 | oseltamivir |
| WV15708 | 1 | 190 | 2 | 182 | oseltamivir |
| WV15825 | 9 | 276 | 16 | 272 | oseltamivir |
| µ | -0,45265 |  |  |  |  |
| sigma | 0,095484 |  |  |  |  |

b

| Study | RR | [95% Conf. | Interval] | % Weight |
| --- | --- | --- | --- | --- |
| 167-101 | 0.488 | 0.124 | 1.915 | 4.30 |
| NAI30034 | 0.175 | 0.061 | 0.504 | 7.17 |
| NAIA3004 | 0.677 | 0.362 | 1.265 | 20.56 |
| NAIA3005 | 0.324 | 0.166 | 0.633 | 17.96 |
| WV15673/WV15697 | 0.416 | 0.259 | 0.667 | 36.00 |
| WV15708 | 0.479 | 0.044 | 5.237 | 1.41 |
| WV15825 | 0.554 | 0.249 | 1.233 | 12.60 |
| D+L pooled RR | 0.432 | 0.325 | 0.574 | 100.00 |

Heterogeneity chi-squared = 5.99 (d.f. = 6) p = 0.424

I-squared (variation in RR attributable to heterogeneity) = 0.0%

Estimate of between-study variance Tau-squared < 0.0001

Test of RR=1 : z= 5.80 p < 0.0001
