# Supplementary material for: Cost-Effectiveness Analysis of Influenza A (H1N1) Chemoprophylaxis in Brazil
Source: Front Pharmacol. 2019 Sep 10;10:945. doi: 10.3389/fphar.2019.00945 (PMC6749104; doi:10.3389/fphar.2019.00945)
Supplement: Supplementary file 2 [file DataSheet_2.pdf]

Supplementary file 2. Critical appraisal of included studies

We used AMSTAR 2 for systematic reviews, Newcastle-Ottawa scale for cohort and case-control studies and the Joanna Briggs Institute checklist for prevalence studies.

Study	Quality assessment tool	Critical appraisal
Jefferson 2014	AMSTAR 2	High quality review
Lau 2012	AMSTAR 2	Critically low quality review
Upjohn 2012	Joanna Briggs Institute checklist	5/9
Carrat 2008	AMSTAR 2	Critically low quality review
Lenzi 2009	Newcastle-Ottawa scale	8/10
Calmona 2013	Joanna Briggs Institute checklist	8/9
Duarte 2009	Newcastle-Ottawa scale	8/9
Hollmann 2013	Newcastle-Ottawa scale	7/10
Zimmermann 2016	Joanna Briggs Institute checklist	8/9
Silva 2017	Joanna Briggs Institute checklist	8/9

The individual assessment of each study is bellow.

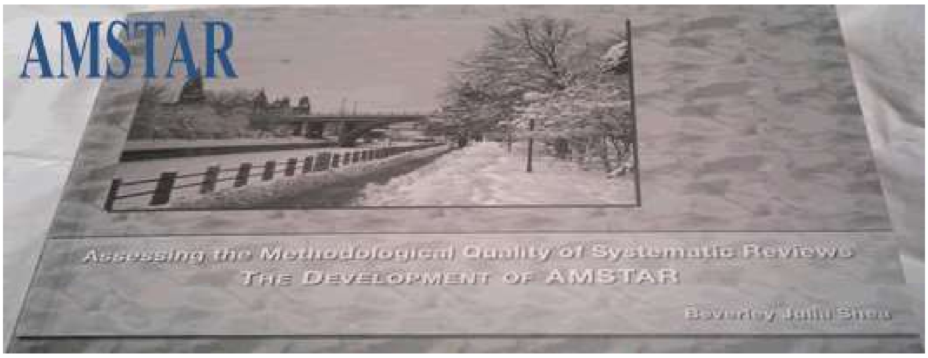

AMSTAR 2 Results

Printer Friendly Version

Article Name: Jefferson 2014

Jefferson 2014 is a High quality review

1. Did the research questions and inclusion criteria for the review include the components of PICO?

Yes
Yes
Yes
Yes
Yes

---

2. Did the report of the review contain an explicit statement that the review methods were established prior to the conduct of the review and did the report justify any significant deviations from the protocol?

YesYesYes

---

3. Did the review authors explain their selection of the study designs for inclusion in the review?

Yes
Yes

---

4. Did the review authors use a comprehensive literature search strategy?

Yes

Yes
Yes
Yes
Yes

---

5. Did the review authors perform study selection in duplicate?

Yes
Yes

---

6. Did the review authors perform data extraction in duplicate?

Yes
Yes

---

7. Did the review authors provide a list of excluded studies and justify the exclusions?

Yes

Yes

---

8. Did the review authors describe the included studies in adequate detail?

Yes

	Yes<br>Yes<br>Yes<br>Yes<br>Yes
**9. Did the review authors use a satisfactory technique for assessing the risk of bias (RoB) in individual studies that were included in the review?**<br>**RCT**	Yes
**NRSI**	0<br><br>Yes<br>Yes
**10. Did the review authors report on the sources of funding for the studies included in the review?**	Yes<br>Yes
**11. If meta-analysis was performed did the review authors use appropriate methods for statistical combination of results?**<br>**RCT**	Yes
**NRSI**	Yes<br>Yes<br>Yes
**12. If meta-analysis was performed, did the review authors assess the potential impact of RoB in individual studies on the results of the meta-analysis or other evidence synthesis?**	Yes
**13. Did the review authors account for RoB in individual studies when interpreting/ discussing the results of the review?**	Yes<br><br>Yes
**14. Did the review authors provide a satisfactory explanation for, and discussion of, any heterogeneity observed in the results of the review?**	Yes<br>Yes
**15. If they performed quantitative synthesis did the review authors carry out an adequate investigation of publication bias (small study bias) and discuss its likely impact on the results of the review?**	Yes<br><br>Yes
**16. Did the review authors report any potential sources of conflict of interest, including any funding they received for conducting the review?**	Yes<br><br>Yes

To cite this tool: Shea BJ, Reeves BC, Wells G, Thuku M, Hamel C, Moran J, Moher D, Tugwell P, Welch V, Kristjansson E, Henry DA. AMSTAR 2: a critical appraisal tool for systematic reviews that include randomised or non-randomised studies of healthcare interventions, or both. BMJ. 2017 Sep 21;358:j4008.

<< Back

Copyright © 2017 AMSTAR All Rights Reserved |

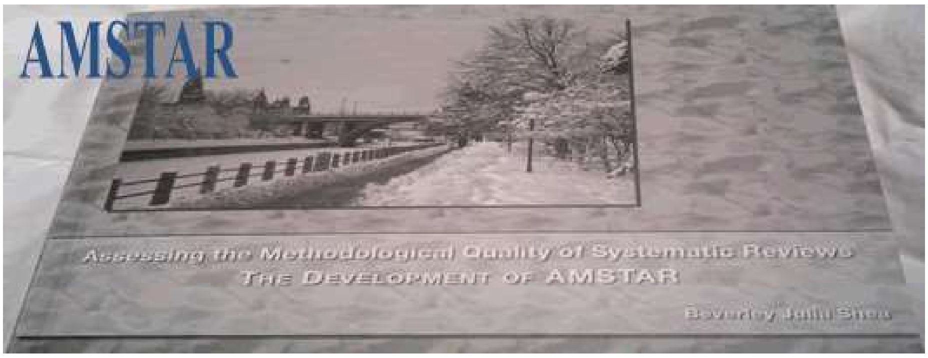

AMSTAR 2 Results

Printer Friendly Version

Article Name: Lau 2012

Lau 2012 is a Critially Low quality review

1. Did the research questions and inclusion criteria for the review include the components of PICO?

Yes
Yes
Yes

Yes

2. Did the report of the review contain an explicit statement that the review methods were established prior to the conduct of the review and did the report justify any significant deviations from the protocol?

YesYes

3. Did the review authors explain their selection of the study designs for inclusion in the review?

No

4. Did the review authors use a comprehensive literature search strategy?

Partial Yes
Yes
Yes
Yes

5. Did the review authors perform study selection in duplicate?

Yes
Yes

6. Did the review authors perform data extraction in duplicate?

No

7. Did the review authors provide a list of excluded studies and justify the exclusions?

Yes

Yes

8. Did the review authors describe the included studies in adequate detail?

Yes

	Yes<br><br><br>Yes<br>Yes
**9. Did the review authors use a satisfactory technique for assessing the risk of bias (RoB) in individual studies that were included in the review?**<br>**RCT**	0
**NRSI**	No
**10. Did the review authors report on the sources of funding for the studies included in the review?**	No
**11. If meta-analysis was performed did the review authors use appropriate methods for statistical combination of results?**<br>**RCT**	
**NRSI**	Yes<br><br><br><br>Yes<br>Yes<br>Yes
**12. If meta-analysis was performed, did the review authors assess the potential impact of RoB in individual studies on the results of the meta-analysis or other evidence synthesis?**	No
**13. Did the review authors account for RoB in individual studies when interpreting/ discussing the results of the review?**	No
**14. Did the review authors provide a satisfactory explanation for, and discussion of, any heterogeneity observed in the results of the review?**	Yes<br><br>Yes
**15. If they performed quantitative synthesis did the review authors carry out an adequate investigation of publication bias (small study bias) and discuss its likely impact on the results of the review?**	No
**16. Did the review authors report any potential sources of conflict of interest, including any funding they received for conducting the review?**	Yes<br>Yes

To cite this tool: Shea BJ, Reeves BC, Wells G, Thuku M, Hamel C, Moran J, Moher D, Tugwell P, Welch V, Kristjansson E, Henry DA. AMSTAR 2: a critical appraisal tool for systematic reviews that include randomised or non-randomised studies of healthcare interventions, or both. BMJ. 2017 Sep 21;358:j4008.

<< Back

Copyright © 2017 AMSTAR All Rights Reserved |

THE JOANNA BRIGGS INSTITUTE

JBI Critical Appraisal Checklist for Studies Reporting Prevalence Data

Reviewer _____Luísa_____________________Date__10/12/2018_____________________________

Author ______Upjohn__________________________Year__2012__Record Number_____________

		Yes	No	Unclear	Not applicable
1.	Was the sample frame appropriate to address the target population?	☐	☐	X	☐
2.	Were study participants sampled in an appropriate way?	X	☐	☐	☐
3.	Was the sample size adequate?	☐	☐	X	☐
4.	Were the study subjects and the setting described in detail?	X	☐	☐	☐
5.	Was the data analysis conducted with sufficient coverage of the identified sample?	X	☐	☐	☐
6.	Were valid methods used for the identification of the condition?	X	☐	☐	☐
7.	Was the condition measured in a standard, reliable way for all participants?	☐	X	☐	☐
8.	Was there appropriate statistical analysis?	☐	☐	☐	X
9.	Was the response rate adequate, and if not, was the low response rate managed appropriately?	X	☐	☐	☐

5/9

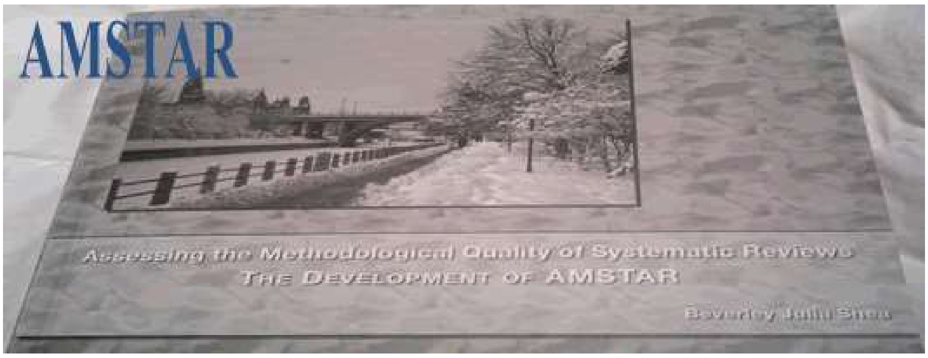

AMSTAR 2 Results

Printer Friendly Version

Article Name: Carrat 2008

Carrat 2008 is a Critially Low quality review

1. Did the research questions and inclusion criteria for the review include the components of PICO?

Yes
Yes
Yes
Yes
Yes

---

2. Did the report of the review contain an explicit statement that the review methods were established prior to the conduct of the review and did the report justify any significant deviations from the protocol?

YesYes

---

3. Did the review authors explain their selection of the study designs for inclusion in the review?

Yes

---

4. Did the review authors use a comprehensive literature search strategy?

Yes

Yes

---

5. Did the review authors perform study selection in duplicate?

Yes
Yes

---

6. Did the review authors perform data extraction in duplicate?

Yes
Yes

---

7. Did the review authors provide a list of excluded studies and justify the exclusions?

No

---

8. Did the review authors describe the included studies in adequate detail?

Partial Yes
Yes

	Yes
**9. Did the review authors use a satisfactory technique for assessing the risk of bias (RoB) in individual studies that were included in the review?**<br>**RCT**	No
**NRSI**	No
**10. Did the review authors report on the sources of funding for the studies included in the review?**	No
**11. If meta-analysis was performed did the review authors use appropriate methods for statistical combination of results?**<br>**RCT**	Yes
**NRSI**	Yes<br>Yes<br>Yes
**12. If meta-analysis was performed, did the review authors assess the potential impact of RoB in individual studies on the results of the meta-analysis or other evidence synthesis?**	No
**13. Did the review authors account for RoB in individual studies when interpreting/ discussing the results of the review?**	No
**14. Did the review authors provide a satisfactory explanation for, and discussion of, any heterogeneity observed in the results of the review?**	No
**15. If they performed quantitative synthesis did the review authors carry out an adequate investigation of publication bias (small study bias) and discuss its likely impact on the results of the review?**	No
**16. Did the review authors report any potential sources of conflict of interest, including any funding they received for conducting the review?**	Yes<br>Yes

To cite this tool: Shea BJ, Reeves BC, Wells G, Thuku M, Hamel C, Moran J, Moher D, Tugwell P, Welch V, Kristjansson E, Henry DA. AMSTAR 2: a critical appraisal tool for systematic reviews that include randomised or non-randomised studies of healthcare interventions, or both. BMJ. 2017 Sep 21;358:j4008.

<< Back

Lenzi 2012 – 8/10 ✵

NEWCASTLE - OTTAWA QUALITY ASSESSMENT SCALE COHORT STUDIES

Note: A study can be awarded a maximum of one star for each numbered item within the Selection and Outcome categories. A maximum of two stars can be given for Comparability

Selection

1) Representativeness of the exposed cohort
   X a) truly representative of the average inpatients influenza (describe) in the community ✵
   b) somewhat representative of the average _______________ in the community ✵
   c) selected group of users eg nurses, volunteers
   d) no description of the derivation of the cohort

2) Selection of the non exposed cohort
   X a) drawn from the same community as the exposed cohort ✵
   b) drawn from a different source
   c) no description of the derivation of the non exposed cohort

3) Ascertainment of exposure
   X a) secure record (eg surgical records) ✵ - SINAN, confirmed with PCR
   b) structured interview ✵
   c) written self report
   d) no description

4) Demonstration that outcome of interest was not present at start of study
   X a) yes ✵
   b) no

Comparability

1) Comparability of cohorts on the basis of the design or analysis
   a) study controls for _____________ (select the most important factor) ✵
   X b) study controls for any additional factor ✵ (This criteria could be modified to indicate specific control for a second important factor.) *The study investigates the risk and protective factors for hospitalization and controls social, treatment, etc.*

Outcome

1) Assessment of outcome
   a) independent blind assessment ✵
   X b) record linkage ✵ - SINAN, interview
   c) self report
   d) no description

2) Was follow-up long enough for outcomes to occur
   X a) yes (select an adequate follow up period for outcome of interest) ✵
   b) no

3) Adequacy of follow up of cohorts
   X a) complete follow up - all subjects accounted for ✵
   b) subjects lost to follow up unlikely to introduce bias - small number lost - > ____ % (select an adequate %) follow up, or description provided of those lost) ✵
   c) follow up rate < ____% (select an adequate %) and no description of those lost
   d) no statement

THE JOANNA BRIGGS INSTITUTE

JBI Critical Appraisal Checklist for Studies Reporting Prevalence Data

Reviewer_____Luisa____________________________Date___________________________________

Author ______Calmona________________________Year___2013___Record Number____________

	Yes	No	Unclear	Not applicable
1. Was the sample frame appropriate to address the target population?	X	☐	☐	☐
2. Were study participants sampled in an appropriate way?	X	☐	☐	☐
3. Was the sample size adequate?	☐	☐	X	☐
4. Were the study subjects and the setting described in detail?	X	☐	☐	☐
5. Was the data analysis conducted with sufficient coverage of the identified sample?	X	☐	☐	☐
6. Were valid methods used for the identification of the condition?	X	☐	☐	☐
7. Was the condition measured in a standard, reliable way for all participants?	X	☐	☐	☐
8. Was there appropriate statistical analysis?	X	☐	☐	☐
9. Was the response rate adequate, and if not, was the low response rate managed appropriately?	X	☐	☐	☐

8/9

Duarte 2009 – 8/9 ✷

NEWCASTLE - OTTAWA QUALITY ASSESSMENT SCALE
COHORT STUDIES

Note: A study can be awarded a maximum of one star for each numbered item within the Selection and Outcome categories. A maximum of two stars can be given for Comparability

Selection

1) Representativeness of the exposed cohort
   a) truly representative of the average _______________ (describe) in the community ✷
   X b) somewhat representative of the average **H1N1 hospital admission** in the community ✷
   c) selected group of users eg nurses, volunteers
   d) no description of the derivation of the cohort

2) Selection of the non exposed cohort
   X a) drawn from the same community as the exposed cohort ✷
   b) drawn from a different source
   c) no description of the derivation of the non exposed cohort

3) Ascertainment of exposure
   X a) secure record (eg surgical records) ✷
   b) structured interview ✷
   c) written self report
   d) no description

4) Demonstration that outcome of interest was not present at start of study
   X a) yes ✷
   b) no

Comparability

1) Comparability of cohorts on the basis of the design or analysis
   X a) study controls for **PCR diagnose** (select the most important factor) ✷
   b) study controls for any additional factor ✷ (This criteria could be modified to indicate specific control for a second important factor.)

Outcome

1) Assessment of outcome
   a) independent blind assessment ✷
   X b) record linkage ✷
   c) self report
   d) no description

2) Was follow-up long enough for outcomes to occur
   X a) yes (select an adequate follow up period for outcome of interest) ✷
   b) no

3) Adequacy of follow up of cohorts
   X a) complete follow up - all subjects accounted for ✷
   b) subjects lost to follow up unlikely to introduce bias - small number lost - > ____ % (select an adequate %) follow up, or description provided of those lost) ✷
   c) follow up rate < ____% (select an adequate %) and no description of those lost
   d) no statement

Hollmann 2013

7/10 ✵

NEWCASTLE - OTTAWA QUALITY ASSESSMENT SCALE
CASE CONTROL STUDIES

Note: A study can be awarded a maximum of one star for each numbered item within the Selection and Exposure categories. A maximum of two stars can be given for Comparability.

Selection

1) Is the case definition adequate?
   X a) yes, with independent validation ✵ *H1N1 confirmed by PCR, inpatient*
   b) yes, eg record linkage or based on self reports
   c) no description

2) Representativeness of the cases
   a) consecutive or obviously representative series of cases ✵
   X b) potential for selection biases or not stated

3) Selection of Controls
   X a) community controls ✵ *H1N1 confirmed by PCR, outpatient*
   b) hospital controls
   c) no description

4) Definition of Controls
   X a) no history of disease (endpoint) ✵ *absence of disease (after the end of infection)*
   b) no description of source

Comparability

1) Comparability of cases and controls on the basis of the design or analysis
   X a) study controls for **hospitalization** (Select the most important factor.) ✵
   b) study controls for any additional factor ✵ (This criteria could be modified to indicate specific control for a second important factor.)

Exposure

1) Ascertainment of exposure
   X a) secure record (eg surgical records) ✵ *PCR*
   b) structured interview where blind to case/control status ✵
   X c) interview not blinded to case/control status *patients aware of the situation, impossible to blind*
   d) written self report or medical record only
   e) no description

2) Same method of ascertainment for cases and controls
   X a) yes ✵ *EQ-5D*
   b) no

3) Non-Response rate
   X a) same rate for both groups ✵
   b) non respondents described
   c) rate different and no designation

THE JOANNA BRIGGS INSTITUTE

JBI Critical Appraisal Checklist for Studies Reporting Prevalence Data

Author Zimmermann _________________________________Year___2016__

	Yes	No	Unclear	Not applicable
1. Was the sample frame appropriate to address the target population?	X	☐	☐	☐
2. Were study participants sampled in an appropriate way?	X	☐	☐	☐
3. Was the sample size adequate?	X	☐	☐	☐
4. Were the study subjects and the setting described in detail?	X	☐	☐	☐
5. Was the data analysis conducted with sufficient coverage of the identified sample?	☐	☐	X	☐
6. Were valid methods used for the identification of the condition?	X	☐	☐	☐
7. Was the condition measured in a standard, reliable way for all participants?	X	☐	☐	☐
8. Was there appropriate statistical analysis?	X	☐	☐	☐
9. Was the response rate adequate, and if not, was the low response rate managed appropriately?	X	☐	☐	☐

8/9

© Joanna Briggs Institute 2017

THE JOANNA BRIGGS INSTITUTE

JBI Critical Appraisal Checklist for Studies Reporting Prevalence Data

Author Silva__________________________________Year 2017

	Yes	No	Unclear	Not applicable
10. Was the sample frame appropriate to address the target population?	X	☐	☐	☐
11. Were study participants sampled in an appropriate way?	X	☐	☐	☐
12. Was the sample size adequate?	X	☐	☐	☐
13. Were the study subjects and the setting described in detail?	X	☐	☐	☐
14. Was the data analysis conducted with sufficient coverage of the identified sample?	☐	☐	X	☐
15. Were valid methods used for the identification of the condition?	X	☐	☐	☐
16. Was the condition measured in a standard, reliable way for all participants?	X	☐	☐	☐
17. Was there appropriate statistical analysis?	X	☐	☐	☐
18. Was the response rate adequate, and if not, was the low response rate managed appropriately?	X	☐	☐	☐

8/9

© Joanna Briggs Institute 2017
